# Fish Health Management Practices and Disease Occurrence in Pond and Cage Aquaculture Systems in the Pwani, Dar es Salaam, and Mwanza Regions, Tanzania

**DOI:** 10.1155/vmi/6596164

**Published:** 2026-07-17

**Authors:** Jestina O. Ntaguda, Aviti J. Mmochi, Amr A. A. Gamil, Stephen Mutoloki, Øystein Evensen

**Affiliations:** ^1^ Institute of Marine Sciences, University of Dar es Salaam, P. O. Box 668, Zanzibar, Tanzania, udsm.ac.tz; ^2^ Faculty of Veterinary Medicine, Department of Paraclinical Sciences, Norwegian University of Life Sciences, P. O. Box 5003, 1432, Ås, Norway, nmbu.no

**Keywords:** aquaculture, cage, diseases, fish health, tilapia

## Abstract

This study assessed fish farms, general fish health management, disease occurrences, and management practices in Tanzania’s pond and cage culture systems. Data were collected using semistructured questionnaires from operational farms across three regions (Pwani, Dar es Salaam, and Mwanza). The questionnaire survey assessed farmers’ demographic data, general pond management practices, and health management issues, including pond treatment, the use of disinfectants and antibiotics, disease and mortality, fish waste management, and net handling. The results showed that the majority (93.2%) of respondents were male, and 34.1% were aged 40–49 years. Most farmers owned ponds/tanks (69.7%), and the remaining owned cages (30.3%). Tilapia was the most farmed fish species (97.0%), followed by the polyculture of tilapia and catfish. Except for the lake, where all cage farms were located, rivers, springs, wetlands, boreholes, and municipal piped water were other sources of water. All water sources for pond‐based farms lacked pretreatment reservoirs. Most fish farms (87/131) had experienced fish diseases and mortalities. Only 20.6% of respondents reported receiving training in fish health and disease management. Most respondents knew that fish could get sick, but training in disease diagnosis was poor. The clinical signs that respondents listed included decreased feed intake, erratic movements, skin ulcers, a distended abdomen, darkened skin, white skin patches, fin rot and scale erosion, stunted growth, and protruding eyes. The survey suggests that training attendance and self‐reported diagnostic capacity were limited among farmers in the surveyed regions. Training and capacity building for farmers in disease diagnosis and management are a way forward for enhancing the sustainability and profitability of tilapia farming in Tanzania.

## 1. Introduction

Aquaculture provides aquatic foods and serves as a source of livelihood in developing countries, including Tanzania [1, 2]. In recent years, global aquaculture development has steadily increased. At the same time, capture fisheries have stagnated, making aquaculture one of the most viable alternatives to address food deficits worldwide [1].

Aquaculture in Tanzania is rapidly moving from extensive to semi‐intensive and intensive farming systems [2, 3]. The intensification of aquaculture has been hindered by infections and mortalities, as well as a lack of high‐quality seeds and feed, which have challenged its development and sustainability [4]. The financial losses and increased production costs due to mortalities, treatment expenses, and reduced growth during recovery discourage would‐be farmers from entering the sector, thereby hampering the development of the sector [4–8]. Hence, implementation of appropriate health management and biosecurity measures is essential for the effective development and sustainability of aquaculture production in Tanzania [8, 9].

Implementation of proper management plans requires a proper understanding and diagnosis of the disease challenges present in the production area. Fish diagnostic laboratories and fish health support services are extremely limited in Tanzania, with most facilities located within universities and government research centers, often far from most rural fish farms [10, 11]. This geographic separation deters local fish farmers from accessing timely and effective diagnostic services. Consequently, many farmers lack adequate knowledge about common fish disease symptoms and are unable to identify early signs of illness in their stocks. In the absence of proper recognition of these indicators, farmers rarely report disease outbreaks or fish mortality events to the relevant authorities [4]. A lack of capacity in fish disease diagnosis, control, and prevention among fisheries and aquaculture extension officers significantly contributes to farmers’ limited knowledge of undertaking biosecurity measures, as well as health management and disease diagnosis [4, 8]. In addition, as aquaculture in Tanzania grows, mortalities due to poor fish health management are becoming common in farms. Therefore, establishing fish disease control and prevention procedures that support the sustainability and expansion of the aquaculture industry is critical [6, 8, 12]. This study assessed the management practices in Tanzanian fish farms and their effects on disease occurrence.

## 2. Material and Methods

### 2.1. Ethical Considerations

The study was conducted in accordance with Tanzanian laws and guidelines for fisheries regulations. Accordingly, all participants were asked to complete the survey voluntarily before the questionnaires [3]. Participants were assured of anonymity and confidentiality throughout the survey process, including the publication of the results.

### 2.2. Study Area

**FIGURE 1:**
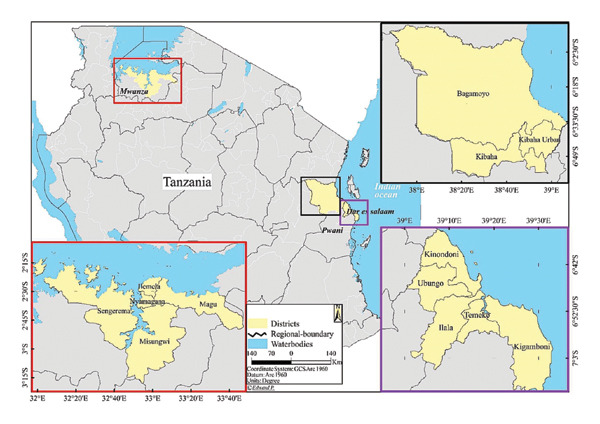
Map of Tanzania showing the study areas in three regions (the lake zone and coastal zone) and 12 districts: Ilemela, Nyamagana, Magu, Misungwi, and Sengerema in Mwanza; (Kinondoni, Kigamboni, Temeke, and Ilala in Dar es Salaam; and (Kibaha, and Bagamoyo in Pwani. Source: from the Institute of Marine Sciences (IMS) database.

The study was conducted from January to May 2023 in the coastal area (Pwani and Dar es Salaam regions) and the lake zone (Mwanza region) on the mainland of Tanzania. Three regions comprising 12 districts were covered (Figure 1).

### 2.3. Study Design and Sampling

A cross‐sectional study design was adopted. Semistructured questionnaires were prepared using the KoBoToolbox software, an online data collection platform available at https://www.kobotoolbox.org/ and installed on Android tablets. A form resembling the developed questionnaire for this study was created and linked to the KoBoCollect app on Android tablets. Before administering the questionnaire, a pilot study was conducted, in which a sample of 10 farms was randomly selected by lottery to participate in interviews to assess the strengths and weaknesses of the developed study tool. The final version, incorporating the pretest results, was used for all subsequent interviews. A list of all fish farmers in a particular region visited was collected from regional/district fisheries officers and accounted for 100% of the sample size. The interviews were conducted face‐to‐face with 131 farmers. The interview was conducted in Kiswahili and English, languages that most farmers use, with the help of enumerators (3 persons). The target respondents were either the farm owners, farm managers, or attendants/caretakers. In the absence of farm owners, managers, or attendants/caretakers, a person responsible for day‐to‐day farm management was interviewed in their place. For farms with multiple owners (e.g., groups), only one member was interviewed to represent the group. The interviews with individual farmers lasted approximately 1 hour.

### 2.4. Data Collection

Data were collected by administering a questionnaire designed to gather information on sociodemographics, farm management practices, fish health management, and biosecurity practices (Table 1). Additional information was collected from aquaculture experts and through field observations guided by checklists to verify some of the information obtained through questionnaires. Interview responses were coded, and the frequency and percentage response scores were calculated. The responses were either binomial (0/1) or ordinal, and details are shown in Supporting Table 1.

**TABLE 1:** Information assessed on sociodemographics, farm management practices, fish health management, and biosecurity practices is shown.

Demographic information	Farm management practices	Fish health management and biosecurity practices
Location	Farming systems	Ability to diagnose disease
Age	Type of fish farmed	Clinical signs
Gender	Types of culture facilities	Fish health training attendance
Education level	Pond/cage size	History of disease incidences
Title of respondents	Stocking densities	Season of disease occurrences
Farm ownership	Pond fertilization and fertilization frequency	History of mortality and mortality rate
	Pond cleaning	Disease management
	Sources of water and exchange frequency	Handling of dead fish and other fish wastes
	Type of feed and feeding frequency	Use of antibiotics

### 2.5. Data Analysis

Data were entered and cleaned using Microsoft Excel 2016 before being exported to Stata Version 18 (StataCorp LLC, Texas, USA) for quantitative data analysis. Qualitative data from the questionnaire were coded to generate quantitative data as described elsewhere [13]. Quantitative data from semistructured questionnaire interviews were analyzed for descriptive statistics (percentages and frequencies) and inferential statistics. Mortality was originally recorded as ordered percentage categories, and because the highest categories contained few observations, it was recoded into three ordered levels: 0%–10%, 11%–30%, and above 30% to provide a more balanced outcome for analysis. Associations between the mortality category (dependent variable) and the explanatory variables were examined using ordered logistic regression. Stocking density and production type were included as categorical variables, while respondent age was included as a continuous variable, scaled by 10‐year increments. Results are presented as odds ratios with 95% confidence intervals.

**FIGURE 2:**
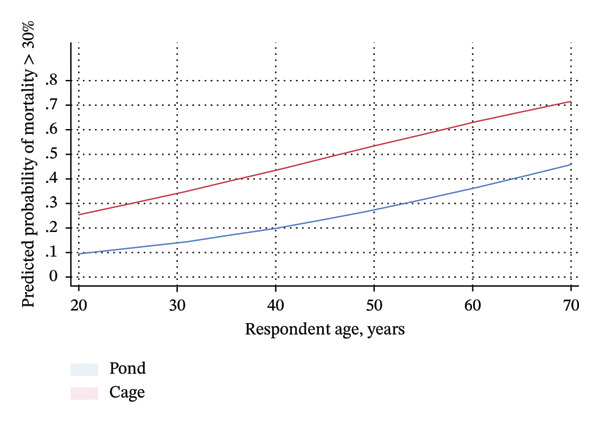
Predicted probability of belonging to the highest mortality category (> 30%) from the ordered logistic regression model, shown by respondent age and production type, adjusted for stocking density.

The linearity of the age effect was assessed by comparing models with and without a quadratic age term using a likelihood ratio test. As the quadratic term did not improve model fit, age was retained as a linear predictor. An interaction between stocking density and production type was explored but not retained because sparse and empty strata yielded unstable estimates. Predictive margins from the final model were used to estimate the adjusted probability of belonging to the highest mortality category. All statistical tests were performed at a significance level of *p* < 0.05. The figures were generated using Matplotlib (in Python) and Stata 18 (Figure 2).

## 3. Results

The results regarding the sociodemographic characteristics of respondents, farm management practices, health management practices, and other challenges are described in detail below.

### 3.1. Sociodemographic Information of Fish Farmers

**FIGURE 3:**
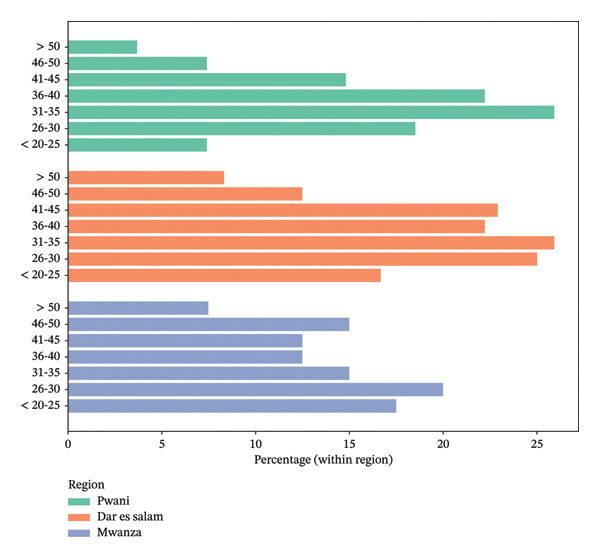
Percentage frequencies of respondents’ age categories by region.

**FIGURE 4:**
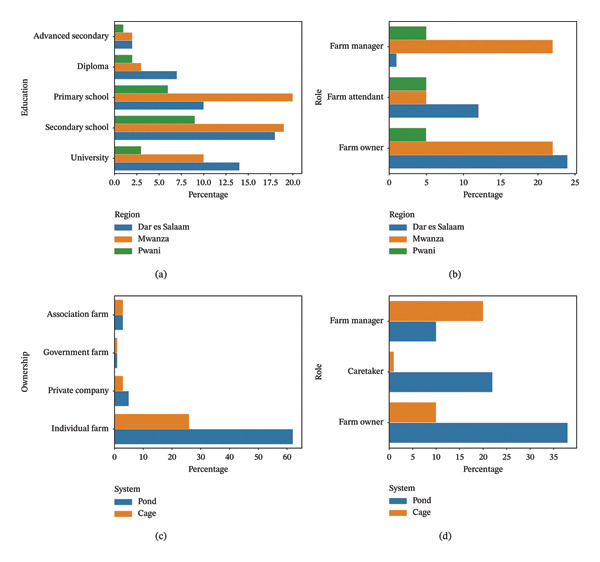
Education levels (a), status of respondents interviewed per region (b), farm ownership per region (c), and farm ownership per culture facilities (d).

The sociodemographic characteristics of respondents are shown in Figures 3 and 4. A total of 131 fish farmers from twelve (12) districts were interviewed, in Pwani (*n* = 19, 14.5%), Dar es Salaam (*n* = 63, 48.1%), and Mwanza (*n* = 49, 37.4%). Most respondents were males (*n* = 122, 93.2%), and only 9 (6.8%) were females. The age distribution of the respondents varied by region (Figure 3a). Most respondents had attained secondary education, followed by diploma, primary, and university education (Figure 4a). Most farms had individual owners (Figure 4c), and the status of the respondents interviewed per region and culture systems was as shown in Figure 4b,d.

### 3.2. General Aquaculture Management Practices in Culture Systems

#### 3.2.1. Type, Ownership, and Number of Culture Facilities

Most farmers (69.7%) owned pond culture systems, the majority in Pwani and Dar es Salaam, including concrete, earthen, pond‐liner, and tank systems, while the remaining 30.3% owned cage culture on Lake Victoria. The number of culture facilities varied among farmers, with the majority owning 1 to 2 cages/ponds (Table 2). The cage size ranged from 25 m^3^ to 60 m^3^, and individual farmers owned most cages (76.9%), while associations and private companies owned the remaining. Fish stocking densities varied among farmers, ranging from 50 to 320 fingerlings per m^3^ in cage culture, and in pond culture, mean fish stocking densities were 20.9 ± 10.8 fingerlings/m^2^.

**TABLE 2:** The majority of the culture facilities owned by individual farmers had fewer than 5 ponds.

Number of ponds/cages	Cage culture		Pond culture	
	Freq.	Percent	Freq.	Percent
1–2	17	43.6	76	83.5
3–5	12	30.8	8	8.8
6–8	2	5.1	4	4.4
9–10	1	2.6	2	2.2
11–30	4	10.3	3	3.3
31+	3	7.7	1	1.1

#### 3.2.2. Type of Farming, Sources of Seeds, Fish Species Farmed, and Water Source for Culture Systems

The monoculture fish farming system for Nile tilapia (*Oreochromis niloticus*) was practiced by the majority of fish farmers (123, 93.1%) in both cage and pond culture; only 8 (6.1%) in pond culture practiced polyculture. Most farms practicing pond farming (71.4%, 65 of 92) reported culturing mixed‐sex tilapia, while the remaining 25.3% reared monosex tilapia. This was different from cage farming, where the majority reared monosex tilapia (76.9%), and 23.1% reared mixed tilapia.

**FIGURE 5:**
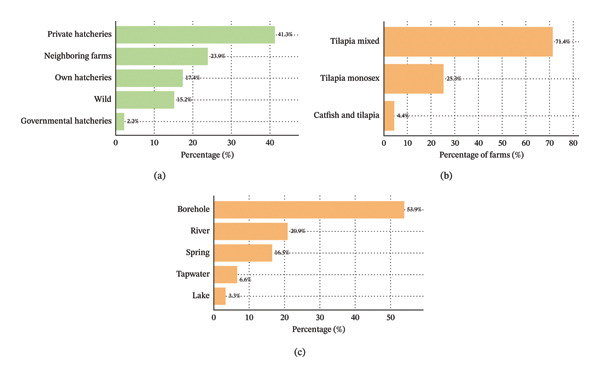
Type of (a) seed sources for fingerlings, (b) fish species farmed, and (c) water source for culture systems.

**FIGURE 6:**
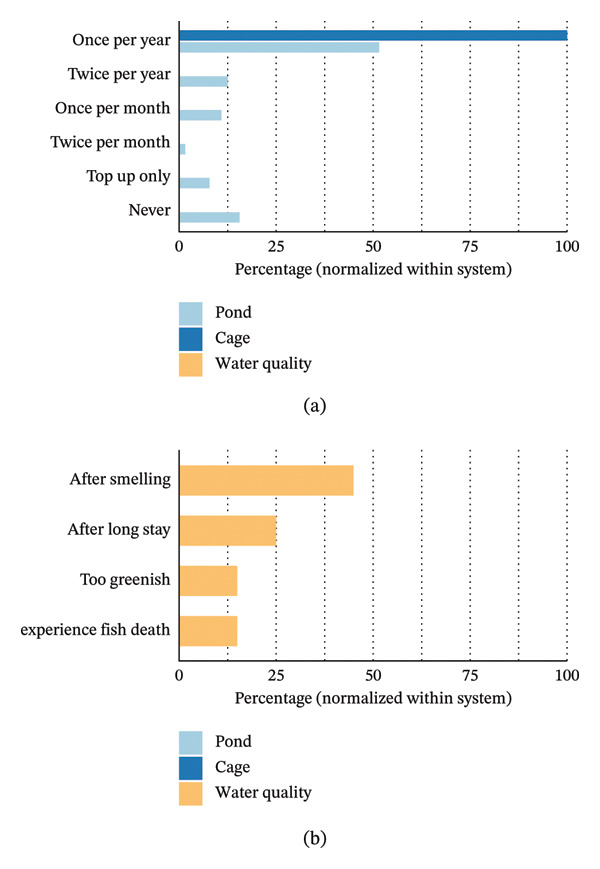
(a) Water exchange frequencies in cages and ponds and (b) motivation to exchange water in pond culture.

Many farmers in cage and pond culture reported obtaining fingerlings from private hatcheries (41.3%), governmental hatcheries (2.2%), neighboring farms (23.9%), and wild (15.2%), and 17.4% had their own hatcheries (Figure 5a). Mixed culture of rearing both Nile tilapia and African catfish (*Clarias gariepinus*) was done in 4.4% (4 of 92) of the pond culture farms (Figure 5b). The farming systems were extensive, semi‐intensive, and intensive. The source of water for all cage culture farms was Lake Victoria, while in pond culture farms, the main sources were boreholes (53.9%), rivers (20.9%), springs/wetlands (16.5%), municipal piped water (6.6%), and (3.3%) Lake Victoria for ponds located in the lake zone (Figure 5c). All the water sources for the pond‐based culture farms lacked pre‐treatment reservoirs before use in the ponds. The majority of farmers replenished water once a year by either topping up or refilling their ponds due to various circumstances (Figure 6a), such as fish mortalities, algae blooms, or prolonged periods where water quality deteriorated (Figure 6b).

#### 3.2.3. Fertilization, Feeding, and Water Exchange Frequency

**FIGURE 7:**
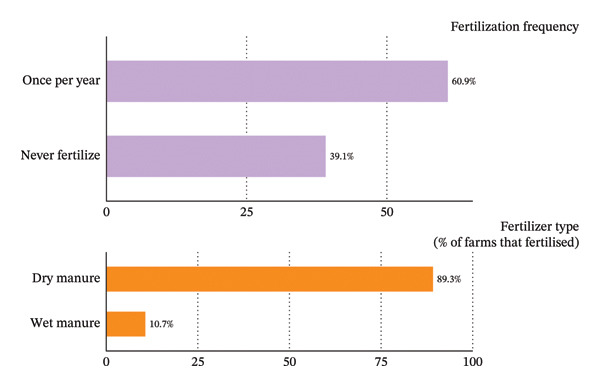
Fertilization frequencies and fertilizer types used to fertilize ponds in pond culture systems.

Most farmers who employed pond culture (*n* = 56) reported regularly fertilizing their ponds with animal manure, such as cow dung, chicken, or rabbit manure, while 39.6% (36) never fertilized their ponds (Figure 7). Among those who fertilized, 89.3% applied dried manure and 10.7% used wet dung (Figure 7). No farmers reported fertilizing farms in cage culture systems.

#### 3.2.4. Feed Type and Feeding Frequency

**FIGURE 8:**
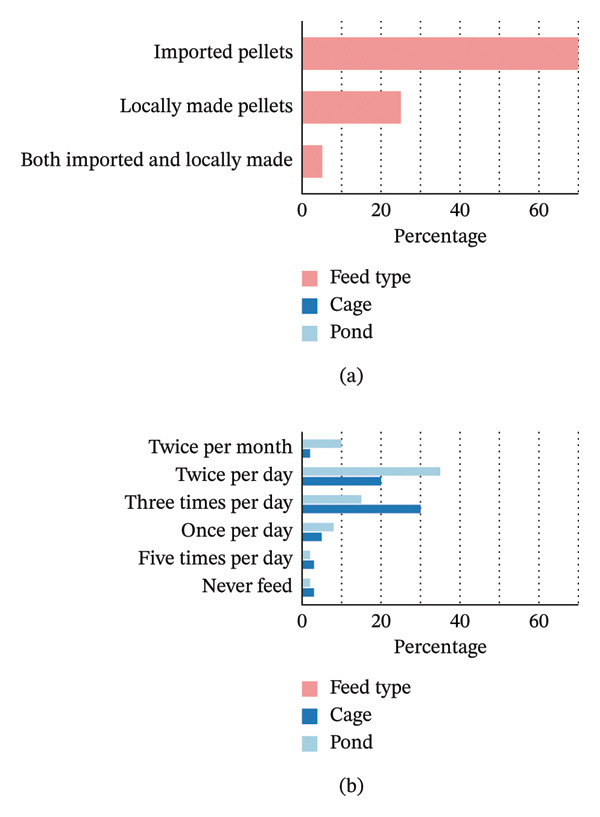
The type of feed used to feed their fish and the feeding frequencies practiced by fish farmers.

Many farmers reported using imported feed pellets, while others used locally made feed pellets (Figure 8a). Feeding frequency varied among cage farms, but most farmers fed their fish three times daily (Figure 8b).

### 3.3. Health Management Practices and Biosecurity Measures

#### 3.3.1. Health Training

**FIGURE 9:**
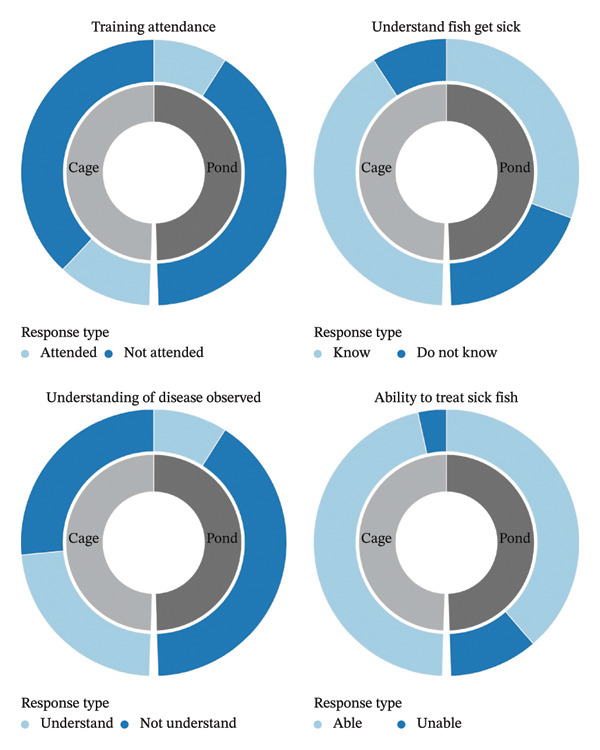
Farmers’ responses to training attendance, understanding of fish getting sick, the disease signs observed, and the ability to treat sick fish.

Most farmers have never attended fish health management training (Figure 9). Of the farmers who had received training, 38.5% received training in general aquaculture and fish health management practices, while the remaining 61.5% received only general aquaculture management practices. The trainings were reportedly conducted by fisheries departments, private fish farms, and nongovernmental organizations (NGOs). Most farmers (89.3%) expressed the need for training in fish diseases and health management practices due to their limited knowledge. The understanding that fish can get sick and the disease signs observed were significantly higher among cage culture personnel (*p* < 0.001), also with a higher ability to treat sick fish (*p* < 0.0001) among these personnel (Figure 9). Only 35.9% of pond fish farmers sought advice from fisheries experts once fish began dying, compared with 74.4% in cage‐culture systems.

#### 3.3.2. Fish Disease Occurrences and Diagnostic Capacity

**FIGURE 10:**
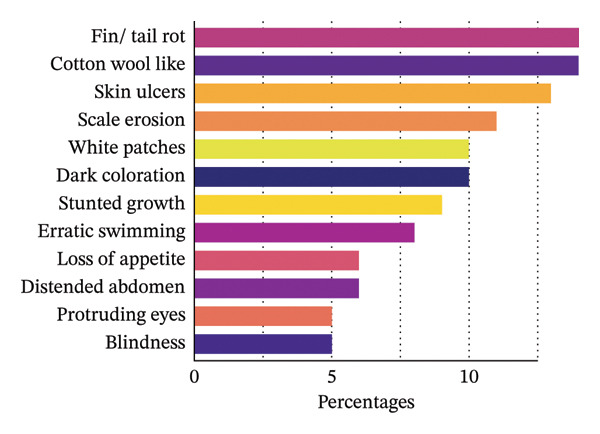
Farmers’ response to the external clinical symptoms recorded in their farms.

Most fish farmers (69.5%, 91/131) reported a history of disease on their farms, but they could not diagnose them. The clinical signs observed and reported by the farmers included cotton‐wool‐like white patches, wounds, tail and fin rot, ulcers, blind eyes, dark skin coloration, abdominal distension, hemorrhages, and erratic swimming (Figure 10). However, among the farmers who experienced disease outbreaks in the areas where questionnaires were administered, only 55.0% of respondents rated fish diseases as a challenge on their farms. More than 25% of the respondents reported disease incidence in the last 6 months, 37.5% in the previous 3 months, and 15.5% at the time of data collection.

#### 3.3.3. Fish Disease Prevention and Treatment

**FIGURE 11:**
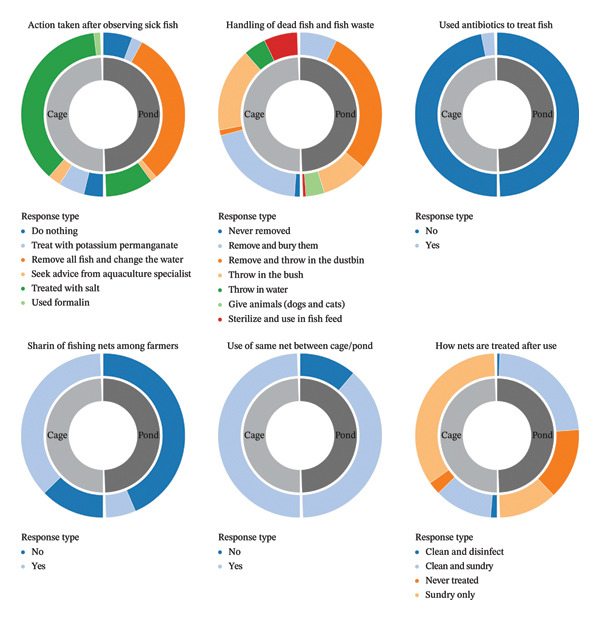
Health management and biosecurity measures practiced by farmers to prevent disease infections on their farms for both cage (left half of the donut) and pond (right half of the donut) culture systems.

Most farmers in both pond and cage culture (98.5%) reported not treating their fish with antibiotics; however, they used lime, salt (sodium chloride), potassium permanganate, copper sulfate, and malachite green to treat bacterial and fungal infections. Only two farmers (1.5%) in cage culture used oxytetracycline to treat bacterial infections, while the other farmers reported using formalin to treat parasites (Figure 11). However, there was no routine administration of those treatments; the majority reported administering the mentioned treatments after experiencing mortalities and disease incidences in their ponds.

#### 3.3.4. Sharing of Nets/Equipment and Net Treatments

Most cage culture farmers reported sharing fishing nets among themselves and between cages, whereas in pond culture, only two farmers reported sharing fishing nets and equipment. Many farmers only sundried their nets after use, and few reported cleaning and disinfecting their nets/equipment before and after use (Figure 11).

#### 3.3.5. Handling of Dead Fish and Fish Wastes

Most pond culture farmers reported disposing of dead fish and fish waste by throwing them into uncovered dustbins, while cage culture farmers primarily disposed of the dead fish in the bushes (Figure 11).

### 3.4. History of Fish Mortality

**FIGURE 12:**
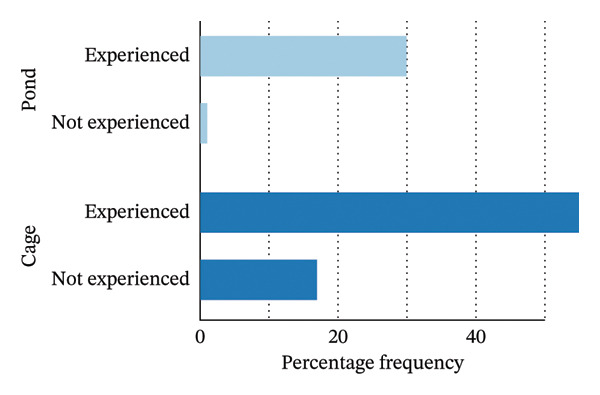
Farmers’ responses to mortality history in pond and cage culture were averaged and normalized within each environment.

Most fish farmers (80.3%, 103/131) involved in pond and cage culture systems had experienced fish mortalities, with a significant difference in mortality between the culture systems (*p* = 0.007); cage culture reported higher mortality than pond culture (Figure 12). The percentage of reported fish mortalities varied between farmers, ranging from 1% to 80% for pond‐stocked fish and 2%–90% in cage culture. Farmers reported mortalities more frequently during the rainy season, in January and February (44.4% for pond and 59.4% for cage), with lower occurrences in March/April (30.6% and 15.9%, for pond and cage, respectively). Lower mortalities for both pond and cage culture systems occurred during the dry season, May to December.

**FIGURE 13:**
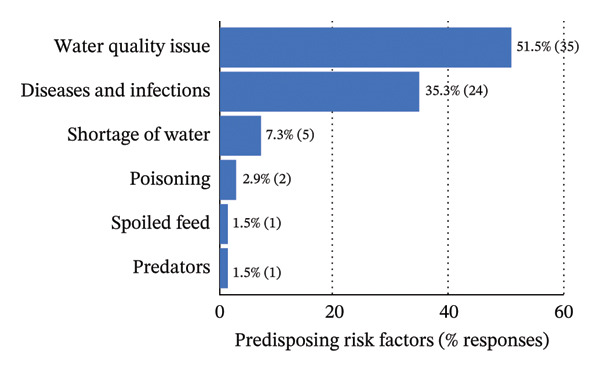
Percentage responses on the predisposing risk factors of fish mortality in pond culture.

**FIGURE 14:**
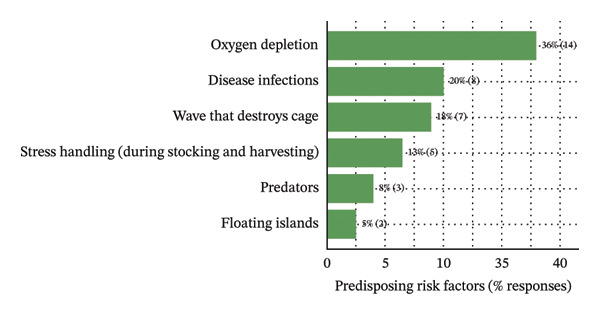
Percentage responses on the predisposing risk factors of fish mortality in cage culture.

Many farmers using pond culture named infections and water quality as predisposing risk factors. In contrast, in cage culture, they identified oxygen depletion and infectious diseases as the primary concerns (Figures 13 and 14).

Next, mortality was recoded into three ordered categories and analyzed using ordered logistic regression. The higher mortality category was associated with stocking density, cage production, and respondent age. Compared with pond farms, cage farms had 3.89 times higher odds of belonging to a higher mortality category. In addition, each 10‐year increase in respondent age was associated with 1.63 times higher odds of belonging to a higher mortality category (Table 3). A likelihood ratio test showed that adding a quadratic age term did not improve model fit, supporting the use of age as a linear predictor. Predicted margins from the ordered logistic model indicated that the probability of belonging to the highest mortality category (above 30%) increased with respondent age in both production systems (Figure 2). Across the full age range, cage farms had a consistently higher predicted probability of high mortality than pond farms (Figure 2).

**TABLE 3:** Ordered logistic regression analysis of factors associated with mortality category in fish farms.

Variable	Category	Odds ratio	95% CI	*p* value
Stocking density	11%–20%	3.31	0.30–36.49	0.329
	21%–30%	51.83	1.73–1552.60	0.023
	31%–40%	8.22	1.22–55.31	0.030
	41%–50%	1.20	0.11–13.54	0.883
	51%–60%	41.69	3.48–499.11	0.003
	> 60%	15.23	1.38–167.85	0.026

Production (culture) type	Cage vs pond	3.89	1.33–11.35	0.013

Respondent age	Per 10‐year increase	1.63	1.02–2.61	0.041

*Note:* Stocking density was included as a categorical variable, as was production type (pond or cage culture) and respondent age (per 10‐year increase). Mortality was categorized into three groups: 0%–10%, 11%–30%, and > 30%. The reference category for stocking density was 0%–10%. Reference categories (base) were stocking density (0%–10%) and pond culture.

## 4. Discussion

This study assessed fish farmers’ management practices in tilapia pond and cage culture in selected regions of Tanzania, with particular focus on fish disease occurrence and mortality. The sociodemographic results indicated that farmers generally had some formal education, with most respondents having completed secondary education, followed by diploma, primary, and university education. This suggests a basic educational foundation that may support improved uptake of training in fish health management, disease recognition, and biosecurity. Most respondents were male, which is consistent with previous studies from Tanzania and neighboring countries showing limited female participation in aquaculture production (Mmanda et al. [14], Mulei et al. [15], and Mzula et al. [8]). They also reported that more male respondents than females engage in aquaculture activities. In many tribal cultures in Tanzania [14], women are expected to perform reproductive roles and take responsibility for household management, food provisioning, and nursing tasks, which hinder their participation in paid economic activities. This also suggests that land ownership is often associated with men owning land and being the primary investors, while women are typically involved later in the process.

General farm management practices varied between fish farmers and culture systems. Monoculture farming was the dominant practice among farmers, with the majority in both pond and cage culture primarily farming Nile tilapia; only 4 out of 131 (3.0%) in pond culture farmed both tilapia and African catfish (*Clarias gariepinus*). This finding was similar to earlier reports [7, 8, 14], which found that most farmers in Tanzania practiced tilapia monoculture.

There was a significant variation in stocking density between fish farms within and between regions. It ranged from 6 to 330 fish per square meter in pond culture systems. In contrast, the cage culture ranged from 36 to 250 fingerlings per cubic meter. This differed from the reported optimum stocking density of 50–120 fish/m^3^ [16–18] in cage culture. Stocking density varied substantially between farms and between production systems. Such variation likely reflects differences in management experience, production objectives, access to seed and feed, and local farming traditions. We found that mortality was associated with stocking density category in the ordered logistic regression analysis, supporting the general view that stocking density is an important determinant of fish health and survival. Although the association was not perfectly monotonic across all density classes, several higher‐density categories were associated with increased odds of higher mortality.

Cage farms had higher odds of belonging to a higher mortality category than pond farms. This is biologically plausible, as increasing density may contribute to crowding stress, impaired water quality, reduced oxygen availability, and enhanced transmission of opportunistic pathogens. At the same time, the wide confidence intervals indicate that the exact magnitude of these effects should be interpreted with caution. Further, the respondent’s age was positively associated with mortality category, the odds increasing by 63% for each 10‐year increase in age. This association should not be interpreted as a direct causal effect of age itself, but rather as a possible indicator of differences in management approach, training history, adoption of updated health practices, or other farm‐level characteristics not captured in the questionnaire. It is possible that younger farmers or farm attendants are more exposed to recent training initiatives or are more willing to adopt new management routines. This finding should therefore be interpreted with caution, but it may still have practical relevance for extension services and training programs.

The water sources for the pond‐based culture farms included rivers, springs, wetlands, boreholes, municipal piped tap water, and lakes. Most farmers had no pre‐treatment reservoirs before using water in ponds. This indicates a potential entry point for pathogens into the ponds from the water source [19]. Water exchanges must be considered when farming fish at higher stocking densities to minimize environmental and physiological stress resulting from poor water quality, such as high ammonia levels, turbidity, and anoxic conditions. The majority of sites exchanged water after certain circumstances, such as a bad smell, being too greenish, experiencing fish death, and after an extended stay. Maintaining good water quality through water exchange is vital for overall fish performance, health, and disease prevention.

Pond fertilization methods were used to increase the supply of natural food organisms for fish and reduce production costs. The manure used was mainly dry cow dung, supplemented by chicken and rabbit manure, while fewer farmers used wet manure. Adding manure, especially wet manure, to fertilize ponds could, in principle, increase the risk of introducing pathogens into the culture system. However, there was no clear indication that using manure fertilization increased the risk of mortalities, similar to findings from other studies [15, 20]

Farmers fed their fish both imported and locally made feed. The majority of farmers fed their fish twice a day for grow‐out fish and three times a day for fingerlings and juveniles. Inconsistent feeding frequency can trigger feeding frenzies, resulting in fish injuries and stress that suppresses immune function and facilitates disease transmission. Moreover, overfeeding leads to uneaten feed accumulating in the water, which decomposes and increases ammonia levels, ultimately reducing dissolved oxygen. Poor water quality stresses weaken fish immune systems, making them more susceptible to disease infections. Therefore, proper feeding practices and the use of quality feeds are recommended for the effective health management of fish in semi‐intensive and intensive cultures [5, 15].

Fish health management refers to the practices designed to prevent fish diseases and infections in aquaculture systems [12]. This study found that most fish farmers have low training attendance and report an inability to diagnose disease, including on‐farm diagnosis based on clinical signs. Only a few farmers in pond‐based culture farms reported being able to observe clinical signs of disease before fish died, compared with cage fish farmers. The reported clinical signs were consistent with external infections and poor fish health, but no etiological diagnosis was confirmed in this survey, aligning with previous reports [8, 19, 21]. The findings indicate gaps in training and reported diagnostic ability. This suggests the need for fish health management training and the creation of awareness among fish farmers in Tanzania.

The fish disease prevention and treatment practices most commonly used in fish farms were the application of salt (sodium chloride and copper II sulfate) and lime. However, most fish farmers in the study areas were unaware of the correct salt concentration to administer. Keeping freshwater fish in water with 5–6 ppt of salt during handling, fasting, and transporting is effective in preventing infections by fungi and external bacteria. However, when fish are already infected, salt baths of 20–30 ppt. for 10–30 min or prolonged baths (10–15 ppt. for 6–12 h) are recommended [22].

Additionally, it is recommended that aeration be maintained to ensure an adequate oxygen supply inside the enclosure during treatment [23–25]. Moreover, a few farmers reported using malachite green, a chemical substance banned for use in aquaculture in the Northern hemisphere due to its teratogenic and mutagenic properties [26]. None in the pond culture reported using antibiotics, and only two farmers in the cage culture reported using antibiotics (oxytetracycline, penicillin, and amoxicillin) when they observed fish deaths, somewhat in contrast to previous findings, where more cage culture farmers and fewer pond culture farmers used antibiotics in disease treatment [19].

## 5. Conclusion

The study demonstrates that fish farmers in the surveyed regions of Tanzania operate predominantly pond‐based culture systems, with cage culture confined to Lake Victoria. Nile tilapia monoculture constitutes the main production practice. Although disease occurrence and fish mortalities were widely reported, training in fish health management and disease diagnosis among farmers was limited. Moreover, biosecurity measures were inconsistently implemented, linked to a limited understanding of general fish health management practices. Therefore, strengthening technical training and improving biosecurity are important to enhance aquaculture development and sustainability. Additionally, further research is recommended to identify and characterize the reported infections and confirm the information gathered in this baseline survey regarding fish diseases. Dissemination of the obtained findings to the public and policymakers is recommended.

## Funding

This study was funded by the Direktoratet for Utviklingssamarbeid, no. QZA‐21/0182.

## Conflicts of Interest

The authors declare no conflicts of interest.

## Supporting Information

Additional supporting information can be found online in the Supporting Information section.

## Supporting information


**Supporting Information 1** Main variables from questionnaire: Supporting Table 1.xlsx.

**Supporting Information 2** Detailed questionnaire overview: Questionnaire_overview.xlsx.

## Data Availability

The data that support the findings of this study are available from the corresponding author upon reasonable request.
